# Performance Evaluation of MPTCP on Simultaneous Use of 5G and 4G Networks

**DOI:** 10.3390/s22197509

**Published:** 2022-10-03

**Authors:** Imtiaz Mahmud, Tabassum Lubna, You-Ze Cho

**Affiliations:** School of Electronic and Electrical Engineering, Kyungpook National University, Daegu 41566, Korea

**Keywords:** multipath TCP, MPTCP, MPTCP schedulers, MPTCP congestion control algorithms, 4G, 5G

## Abstract

The 5G cellular network comes with a promise to provide a very high data rate at low latency, which is becoming critical for advancing technologies. Mobile operators are currently deploying the 5G cellular network worldwide. However, because of limited coverage and high susceptibility of the 5G network to obstacles, handoffs from 5G to 4G and vice versa frequently occur, especially when the user equipment (UE) is moving. These handoffs often cause significant delays in data transmission due to packet losses and retransmissions. A promising solution can be to use both 4G and 5G networks simultaneously, which can solve this problem and yield a better throughput. Multipath transmission control protocol (TCP) is an effective solution for this problem, but it requires significant performance evaluation before practical deployment. In this study, we implement an MPTCP testbed based on NS3-DCE that enables to test the performance of MPTCP schedulers and congestion control algorithms (CCAs) in both 3GPP and non-3GPP networks. Through extensive simulation experiments in a scenario where a UE simultaneously utilizes both 4G and 5G networks, we found that blocking estimation (BLEST) scheduler implemented with balanced linked adaptation (BALIA) CCA can produce the highest throughput and lowest delay. Finally, we showed how received signal to interference and noise ratio (SINR), congestion window, throughput, and packet losses are interconnected.

## 1. Introduction

The worldwide implementation of the 5G cellular network has been opening a new era of communication, facilitating high data transfer speed and low latency compared to previous mobile generations such as 4G [1]. The 5G network has been evolving, focusing on three major supports: enhanced broadband mobile, low latency and ultra-reliable communications, and massive integration for machine communications [2,3]. The upcoming 5G cellular network deployment will bring an estimated USD 13.1 trillion-worth of business globally [4].

The still deploying 5G network leaves many places out of the network coverage, leading to frequent handovers to 4G network and vice versa [5]. Moreover, 5G mmWave signals are comparatively more susceptible to obstacles. Sometimes, even penetrating through a house can substantially reduce its signal strength [6]. This problem yields significant packet losses and more frequent handoff between 5G and 4G networks [7,8,9]. From the transport layer’s point of view, these handoffs can potentially cause significant delay and low throughput in data transfer in the event of reliable data delivery [10,11,12,13]. According to Li et al. [14], transmission control protocol (TCP), which is the key transport layer protocol responsible for reliable end-to-end communication, frequently comes across spurious retransmission timeouts (RTOs) for such handovers causing drastically degraded performance in terms of delay and throughput.

**Figure 1:**
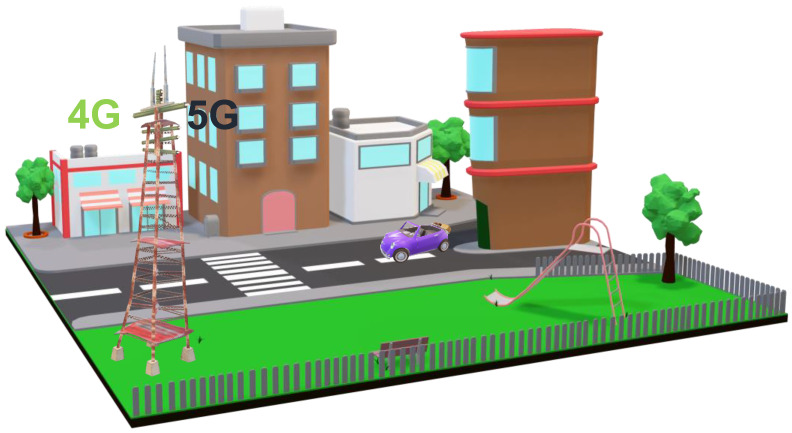
An illustration of the non-standalone implementation of 5G network infrastructure where 5G and 4G base stations are located at the same tower.

Nowadays, most modern devices come with multiple communication interfaces (e.g., 4G, 5G, WiFi, etc.). Generally, they use any one from these available communication interfaces for data communication. Instead of using a single interface at a time, simultaneous use of multiple communication interfaces is expected to provide better performance in terms of throughput and delay, especially in the case of large file transfers [15]. Moreover, keeping the communication up through all the communication interfaces can be a better solution for resolving the spurious RTO problem experienced during the 4G/5G handoffs. On the other hand, most mobile operators are deploying their 5G infrastructure along with their existing 4G infrastructure to reduce costs. Even in a large number of instances, the 4G and 5G antennas are installed in the same tower in a non-standalone (NSA) setup [16], making both the networks available to the user equipment (UE), as can be seen in Figure 1. A simultaneous communication through both the 4G and 5G network interfaces can be a promising solution to produce a better throughput [17]. While the 5G access traffic steering, switching, and splitting (5G ATSSS) focuses on extending support for non-3GPP (third generation partnership project) access networks (e.g., WiFi) to access the 5G core [18,19], following [17], it can be highly effective for the UE to concurrently use both 4G and 5G radios for obtaining high throughput, especially while worldwide full 5G implementation still needs significant time [20].

Multipath TCP (MPTCP) is the modified version of TCP that enables a device to use multiple communication interfaces at the same time [21]. The scheduler and congestion control algorithm (CCA) are the two major parts of MPTCP that mainly controls which path a data packet travels and how much data can be sent on a path, respectively [22]. The key responsibility of the scheduler is to remove head-of-line (HoL) blocking at the receiver, while the CCA is responsible for the maximum utilization of an available path. Together, they try to fulfill the aim of providing high throughput with low latency, which is the ultimate goal of end-to-end communication.

MPTCP can be a promising solution for utilizing both 4G and 5G paths at the same time while resolving spurious RTO problems [14]. Similarly, as previously indicated, combining both 5G and 4G networks concurrently should deliver a higher throughput than using a single path, according to Qualcomm [17]. However, currently, a handful of MPTCP schedulers and CCAs are available, making it difficult to set which combination of scheduler and CCA best suits to resolve the existing problems and yield a better throughput with reduced delay. Moreover, Ding et al. has shown that applying MPTCP over 4G and 5G does not always improve the overall performance [23]. Hence, a proper investigation is necessary before practical deployment.

In this paper, we aim to develop a simulation testbed that will allow us to evaluate the performance of MPTCP in 4G and 5G network situations. Then, using extensive simulation tests, we intend to discover an appropriate combination of MPTCP scheduler and CCA to increase performance in terms of throughput and latency, particularly while using 5G and 4G network interfaces simultaneously. The following are the primary contributions of this paper:

- We developed a simulation testbed using NS3-DCE [24] with MPTCP Linux Kernel v0.90 [25] and NS-3.33 mmWave module [26]. As a result, evaluating the performance of MPTCP on 3GPP (e.g., 5G and 4G) and non-3GPP (e.g., WiFi, WiMax, Ethernet, etc.) networks is possible. For a productive comparison, we also incorporated cutting-edge schedulers, such as redundant, earliest completion first (ECF), and blocking estimation (BLEST), in the MPTCP Linux Kernel v0.90 [25].
- We tested several MPTCP schedulers in combination with available CCAs in MPTCP Linux Kernel, compared their performance in terms of throughput and flow completion time (FCT), and outlined the best combination for real-world deployment.
- During the experiments, we also found that the signal to interference and noise ratio (SINR) directly impacts the obtained throughput of each path. Through proper investigation, we outlined the relation between SINR, throughput, congestion window (CWND), and packet losses; how they impact the performance of different MPTCP schedulers and CCAs.

The rest of the paper is organized as follows: Section 2 presents a summary of the tested MPTCP schedulers and CCAs, Section 3 briefly describes the testbed setup and illustrates the considered simulation scenario for the performance comparison, Section 4 provides a brief analysis of the results outlining the better candidates, and finally, Section 5 concludes the paper.

## 2. An Overview of The MPTCP Schedulers and Congestion Control Algorithms

MPTCP is an extension of TCP that enables simultaneous data transfer through multiple communication interfaces. Each data transfer path between the end devices (i.e., sender and receiver) is regarded as subflow (SF). MPTCP schedulers and CCAs are the two key elements to ensure balanced network sharing, reduced latency, and high throughput. This section briefly describes some existing key MPTCP schedulers and CCAs.

### 2.1. MPTCP Schedulers

MPTCP schedulers are mainly responsible for ensuring in-order delivery at the receiver. Among the available SFs, an MPTCP scheduler decides through which SF data should be sent so that no HoL blocking occurs at the receiver. We tested with five widely accepted MPTCP schedulers, as discussed followingly.

#### 2.1.1. Shortest RTT First

Shortest RTT first (SRTT) sends packets through the SF having the lowest round-trip-time (RTT) among all the SF having an available CWND. CWND is a parameter decided by the CCA that determines how much data can be sent through a SF. When more than one SF has the same lowest RTT, SRTT systematically chooses an SF and continues sending through that SF until the CWND of that SF becomes full. Once the CWND of that chosen SF becomes available again, SRTT starts sending through that SF. SRTT is currently the default scheduler for the MPTCP Linux Kernel [27].

#### 2.1.2. Round-Robin

In the Round-robin scheduler, the SF for sending a packet is selected in a Round-robin fashion [28]. It circularly picks up an SF among all the SFs having available CWND.

#### 2.1.3. Redundant

This scheduler sends the same data through all SFs with space in CWND [29]. This approach of sending the same data through multiple communication interfaces helps reduce the HoL blocking at the receiver, thus ensuring low data delivery latency. However, sending the same packet wastes a significant bandwidth (BW) which could be utilized for sending other essential data by implementing an intelligent scheduling scheme.

#### 2.1.4. Earliest Completion First

The earliest completion first (ECF) scheduler tries to make an intelligent scheduling decision by considering the RTT of the SFs, their available CWND, and the size of the data to be sent [30]. In an abstract form, for making the scheduling decision, it tries to estimate the arrival time of the packets at the receiver and chooses an SF so that the packets arrive in order at the receiver.

#### 2.1.5. Block Estimation

Block estimation (BLEST) scheduler implements a mechanism to predict HoL blocking beforehand [31]. Based on the RTT and send window of an SF, it decides whether sending data through that SF might cause HoL blocking at the receiver or not. If it finds that possibility, BLEST pauses sending data through that SF for some time. In simple form, it waits for the lowest RTT SF to become available even though other SFs might have space in their CWND to avoid HoL blocking at the receiver.

### 2.2. MPTCP Congestion Control Algorithms

MPTCP CCAs are responsible for equally and efficiently sharing the available network resources while ensuring better throughput, fairness, and less delay in data communication. There are three primary design goals for MPTCP CCAs as follows [32]:

- Goal One: MPTCP CCAs should always ensure incentives over single path TCP (SPTCP), implying that the throughput of MPTCP should be at least equal to or greater than SPTCP’s throughput traveling through the same path.
- Goal Two: When multiple MPTCP SFs go through the same bottleneck, they should combinedly occupy a capacity equal to the capacity an SPTCP occupies while going through the same bottleneck.
- Goal Three: After fulfilling the first two goals, MPTCP CCAs should try to move the traffic from the more congested path to a less congested path among the available SFs.

Several MPTCP CCAs have been proposed and made available in the Linux Kernel. The key MPTCP CCAs available in Linux Kernel are discussed as follows.

#### 2.2.1. Linked Increase Algorithm

Linked increase algorithm (LIA) was proposed by Raiciu et al. to fulfill the three design goals of MPTCP [33]. It maintains separate CWND for each SF, increases the CWND on an SF upon reception of acknowledgments (ACKs) on that SF, and decreases it for packet drops. The increase and decrease in CWND of an SF depend on the CWND and RTT of all the SFs. LIA provides a trade-off between optimal congestion balance and responsiveness. LIA obtains a reasonable resource pool by choosing the appropriate amount of increase and decrease in CWND. At the same time, it ensures fairness with SPTCP even without explicitly knowing which SFs are going through a common bottleneck. However, the fairness-ensuring mechanism sometimes behaves so aggressively that LIA obtains a much lower throughput than that of a SPTCP, thus failing to fulfill Goal One, as reported by Lubben et al. [34]. Again, at times, LIA behaves highly aggressively towards SPTCP while not producing a visible advantage for MPTCP, according to Khalili et al. [35].

#### 2.2.2. Opportunistic Linked Increase Algorithm

While LIA provides a trade-off between optimal resource pooling and responsiveness, the opportunistic linked increase algorithm (OLIA) provides both simultaneously [35]. The CWND decrease part of both LIA and OLIA are the same. OLIA adapts Kelly and Voice’s algorithm to provide optimal resource pooling in the increase mechanism [36]. To ensure first responsiveness and non-flappiness, it measures the number of bytes transmitted since the last loss event and reacts to the loss event within the current window. However, OLIA becomes unresponsive to sudden network changes from time to time in a dynamically changing network scenario [37]. Moreover, as OLIA also bases its fairness mechanism on that of LIAs, OLIA also faces similar fairness issues like LIA [34].

#### 2.2.3. Balanced Linked Adaptation

Through a fluid model analysis, balanced linked adaptation (BALIA) tries to find the existing problems in the previous MPTCP CCAs, such as LIA and OLIA, and attempts to resolve them by providing a better balance among responsiveness, fairness, and flappiness. Thus, BALIA is expected to perform better than the LIA and OLIA. However, according to Peng et al. [37], the same fairness problem observed in LIA and OLIA also exists in BALIA.

#### 2.2.4. Weighted Vegas

Cao et al. [38] proposed weighted Vegas (wVegas) with the aim of fulfilling their proposed “congestion equality principle” rather than IETF’s proposed MPTCP design goals [32]. They proposed a mechanism to monitor packet queueing delay and regarded it as a congestion signal. Then, based on their “congestion equality principle,” they obtained a fine-grained load balancing. However, as they solely focus on fulfilling their proposed “congestion equality principle”, wVegas fails to comply with the MPTCP design goals. Moreover, wVegas shows tremendously lower throughput than SPTCP because of its highly aggressive fairer nature.

### 2.3. Existing Research on Performance Analysis of MPTCP Schedulers

Several research papers have been published that focus on the performance analysis of MPTCP schedulers under various scenarios. However, most of them did not consider scenarios considering 5G networks. In MiniNet [39] emulation experiments, different schedulers’ performances were compared in a basic scenario where the sender and receiver send the packets through two different paths [40]. Thakur et al. [41] implemented that same scenario in a testbed and tested different schedulers’ performance. Sathyanarayana et al. [42] exploited the MPTCP schedulers’ performance in their testbed where the client connects to the server via two different cellular network operators. Kumar et al. [43] compared the performance of different schedulers under the 5G/B5G hybrid network in a physical testbed. However, they did not consider mobility when observing the schedulers’ performance while the UE is on the move. Table 1 summarizes the key considerations of these research works. Moreover, in [44], Prakash et al. tested the fidelity of the available NS3-DCE [24] simulator for MPTCP with MPTCP testbed results. However, to the best of our knowledge, no research work has tried to enable an NS3-DCE-based 5G MPTCP simulator that can help researchers understand the performance of MPTCP schedulers in a 5G network scenario, even when the UE is on the move. This is very vital for future research to grasp a proper estimation of MPTCP’s performance in 5G networks.

## 3. Experimental Setup

This section briefly describes the experimental setup for comparing the performance of the considered MPTCP schedulers and CCAs. We briefly describe the testbed setup process combining NS3-DCE [24], MPTCP Linux Kernel v0.90 [25], and NS-3.33 mmWave [26] module. Then, we discuss the considered experimental scenario.

### 3.1. Testbed Setup

We prepared the testbed in a VirtualBox [45] virtual machine with Ubuntu 16.04 LTS [46] operating system. First, we downloaded the NS3-DCE [24] using bake. Then, we modified the bake configure file so that NS3-DCE downloads NS-3.33 [47] instead of the latest version of NS-3. Then, we replaced the downloaded NS-3.33 directory’s files with the NS-3.33 mmWave [26] module’s files. We modified some functions’ code in “model/linux/ipv6-linux.cc” and “model/linux/ipv6-linux.h” files in the NS3-DCE directory so that the NS-3.33 mmWave module worked properly with NS3-DCE. Then, we configured and compiled NS-3.33 mmWave module using waf. Next, for downloading MPTCP Linux Kernel to work in NS3-DCE, we configured bake to download net-next-libos with MPTCP Linux Kernel v0.90 [25], which is the most recent NS3-DCE compatible MPTCP Linux Kernel. We configured the kernel to install the MPTCP path manager, schedulers, and CCAs. Additionally, we recoded the Redundant, ECF, and BLEST schedulers available in the newer version of MPTCP Linux Kernel to work in MPTCP Linux Kernel v0.90. Then, we compiled the kernel and configured it to work with NS3-DCE. Finally, we configured NS3-DCE using waf so that it successfully recognized all the changes in the modules and was ready to conduct the simulation experiments. For interested readers, we have uploaded the testbed files available at [48].

### 3.2. Simulation Environment

As stated earlier, we want to observe the performance of MPTCP schedulers and CCAs in an environment where the UE is connected to both the 5G and 4G networks. Similarly, we want to see how the MPTCP scheduler and CCAs behave when the network condition (i.e., the signal strength) changes in real-time.

**Figure 2:**
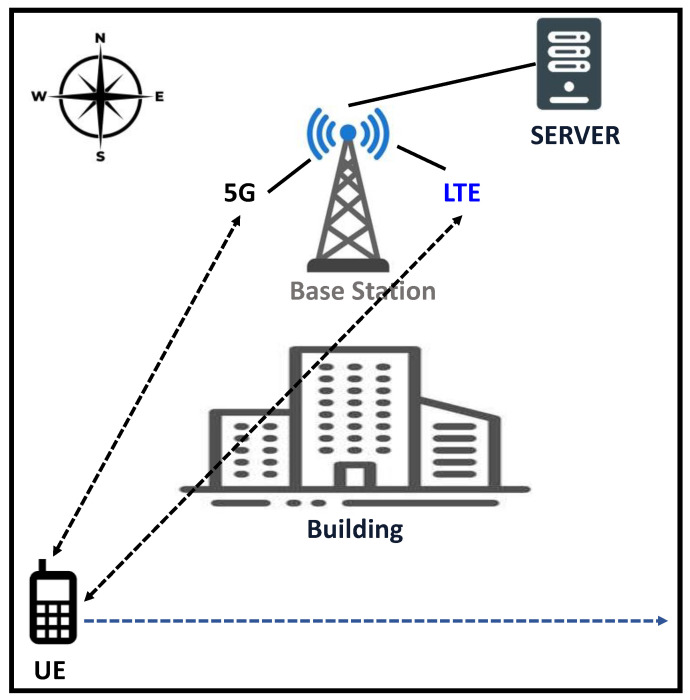
An illustration of the considered simulation scenario.

To simulate such a situation, we consider a scenario where the 5G and 4G base stations are located at the same tower in a non-standalone setup. As shown in Figure 2, the 4G/5G base station is located at the north-mid point of the simulation scenario. Here, the 5G base station offers the 5G mmWave [49] data connection, while the 4G base station offers the 4G Long-Term Evaluation (LTE) [50] connection. To keep the experiments close to practical experiences, we set the uplink and downlink peak rates for 5G as 200 Mbps and 100 Mbps, respectively. Similarly, we considered the uplink and downlink peak rate for 4G as 100 Mbps and 50 Mbps, respectively. The UE moves from the south-west corner of the simulation scenario towards the south-east corner at a 60 km/h speed. There is a house located at the south-mid point of the simulation scenario. At some point in the simulation experiment, while the UE is moving from the south-west corner to the south-east corner, the house comes as an obstacle in the line-of-sight between the base station and UE. As a result, the 5G signal drops when the line-of-sight communication is lost due to the house because of the susceptibility of the 5G signal to obstacles.

We mainly run two types of tests to observe the total amount of received data during the simulation experiment, throughput, and FCT. For observing the total received data and throughput, iperf data transfer takes place for 20 s. To observe the FCT, we repeatedly send a fixed 10-megabyte data for the 20 s simulation time using iperf and take the average FCT. For further investigation and to keep track on the signal strength received by the UE, we record the SINR received by the UE during the simulation time. Furthermore, with the aim of finding a relation among SINR, throughput, and packet losses, we record the number of sent and received protocol data units (PDUs) by the server (*serTx*) and the UE (*ueRx*) during each second of the simulation experiment, respectively. It should be noted that for MPTCP, PDU refers to MPTCP segments. Then, we calculate the difference (Δ*PDU*) by subtracting *serTx* from *ueRx*. From Δ*PDU*, we can infer the received and lost number of MPTCP segments, which can also be regarded as the data packets for the transport layer. A negative and positive value of Δ*PDU* represents the number of lost and recovered PDUs, respectively. For each of the test cases, we run at least 30 experiments and present the average results.

## 4. Performance Evaluation

We investigated the performance of the considered MPTCP scheduler for each of the contemplated MPTCP CCAs. We present the results in brief in this section.

### 4.1. Observed SINR

**Figure 3:**
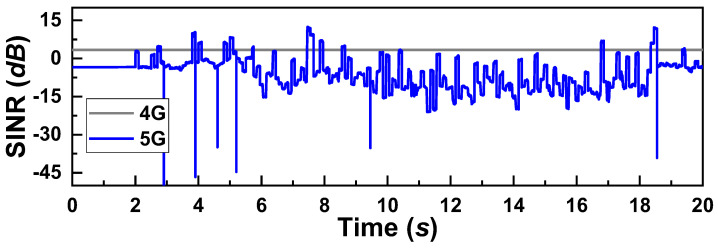
Measured SINR for a test simulation experiment.

As mentioned, we run several experiments for each test case. During the experiments, UE moves the from south-west corner to the south-east corner of the simulation scenario at a 60 km/h speed, and the positions of the 5G/4G base station and the house were fixed. Therefore, an almost identical SINR is observed for all the test cases. Figure 3 shows the SINR observed during a sample test experiment.

### 4.2. Performance Evaluations of MPTCP Schedulers When the CCA Is LIA

**Figure 4:**
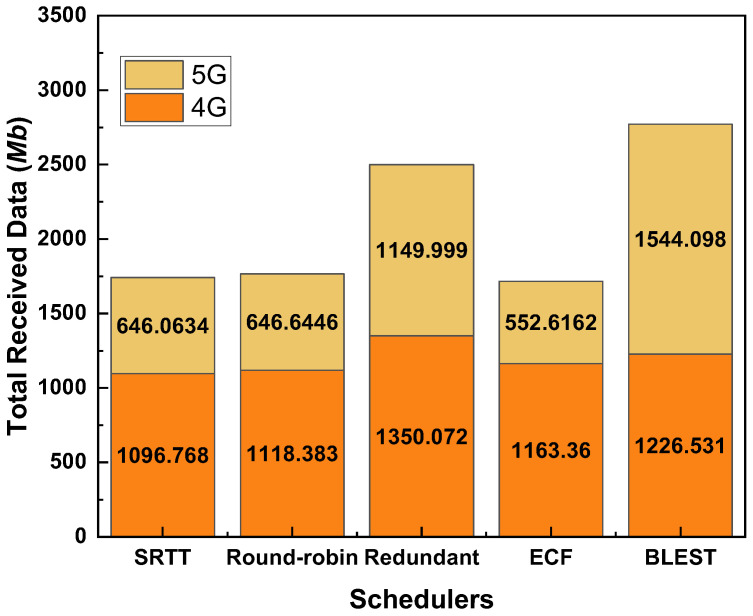
Performance of the tested MPTCP schedulers in terms of total received data for LIA as a CCA.

Figure 4 shows the total received data for the considered MPTCP schedulers while the CCA is LIA. As we can observe, BLEST performs the best among the tested MPTCP schedulers. Redundant scheduler performs the second best. SRTT, Round-robin, and ECF perform almost similarly. As we know, Redundant scheduler sends the same data through all the available paths. This helps reduce the out-of-order delivery of packets at the receiver and, ultimately, helps to eliminate HoL blocking. That is the key reason behind the better performance of the Redundant scheduler than SRTT, Round-robin, and ECF. Blest scheduler also implements the subflow blocking mechanism targetting the elimination of HoL blocking at the receiver. This mechanism proves to be more efficient even than the Redundant one. In Blest, when it estimates that the possibility of HoL blocking at the receiver is low, it sends packets through all the available paths. In contrary, when it estimates that there is a high possibility of HoL blocking at the receiver if packets are sent through a specific SF, it temporarily stops sending via that SF. In this way, it ensures sending more data packets on the one hand and significantly reduces the HoL blocking on the other hand. In support of this hypothesis, we can observe that BLEST could best utilize the 5G network by receiving the highest amount of data via it. This implies that when a stable 5G network is available, BLEST could take the best performance out of it. Combinedly with the 4G network, BLEST could successfully attain the highest amount of received data.

**Figure 5:**
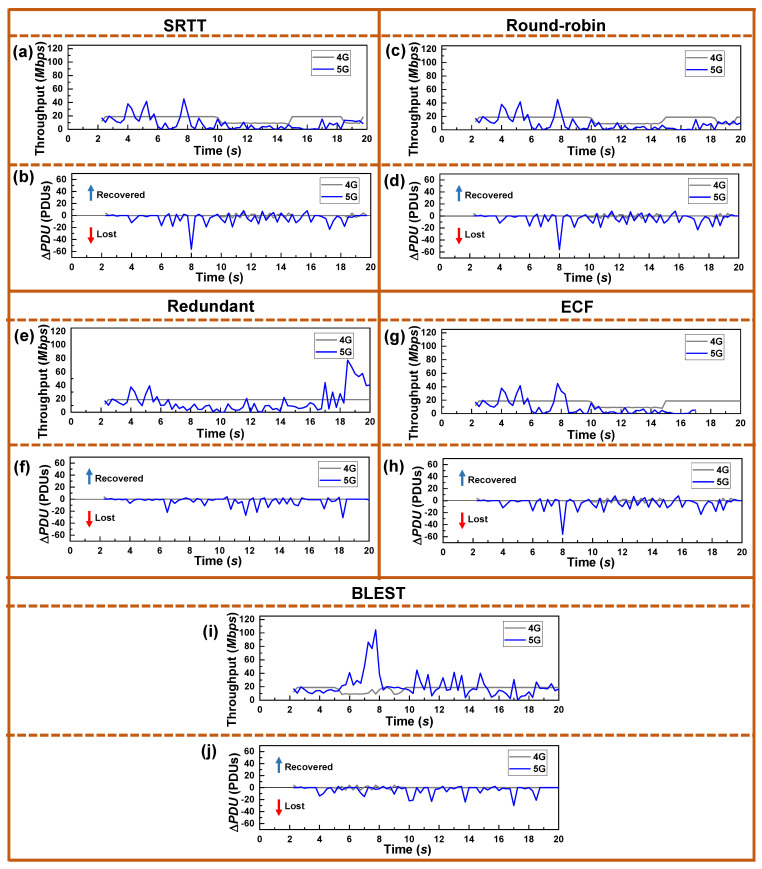
Real-time performance of the tested MPTCP schedulers for LIA as a CCA in terms of throughput and Δ*PDU* for: (**a**,**b**) SRTT, (**c**,**d**) Round-robin, (**e**,**f**) Redundant, (**g**,**h**) ECF, and (**i**,**j**) BLEST, respectively.

Now, to further investigate how the schedulers are operating in real-time, we plot the throughput and Δ*PDU* observed at the UE in Figure 5. Note that, aiming at the proper comparison of the results, we present the results of the experiments where the received SINR was the same as in Figure 3. Here, by having a close observation, we can find a direct relationship between the throughput, Δ*PDU*, and SINR (Figure 3). For example, let us closely observe the spike on the Δ*PDU* at the 8-second time for SRTT, Round-robin, and ECF schedulers. As already mentioned, such a negative spike indicates significant packet losses. We can observe that, right before the 8-second time, the 5G signal’s SINR (Figure 3) improves. At the same time, throughput also increases. This indicates that when the 5G SINR grows, the UE receives the ACKs for the previously sent packets through the 5G SF, resulting in better throughput. By receiving the ACKs, the CCA, which is the LIA in this experiment, increases the CWND of that SF. As the CWND is available and 5G offers the lowest RTT, SRTT, Round-robin, and ECF schedulers send data through this SF. However, the SINR drops at that time, resulting in a loss of packets, which is indicated by the sharp negative spike. Ultimately, this causes a significant HoL blocking at the receiver and a substantial reduction in throughput. As the Redundant scheduler sends the same data through both the SFs, it could avoid such drastic packet losses and reduction in throughput. Due to the intelligent HoL estimation and SF blocking mechanism, BLEST could also avoid such a situation and obtain a considerably better throughput.

Finally, Table 2 summarizes the observed FCT for the tested MPTCP schedulers. Similar to the observed throughput, the FCT of BLEST and Redundant schedulers are significantly lower than that of the SRTT, Round-robin, and ECF. BLEST could complete the flows in the shortest possible time with an average of 7.219 ms. ECF takes the highest time with an average of 11.655 ms. It is worth mentioning that, although ECF implements a well intelligent scheduling mechanism too, we believe its failure to obtain a good outcome is due to its inability to adapt to the rapidly changing nature of the 5G network. The key reason behind the rapid changes in the 5G network is its susceptibility to obstacles, which results in rapid changes in its SINR.

### 4.3. Performance Evaluations of MPTCP Schedulers When the CCA Is OLIA

**Figure 6:**
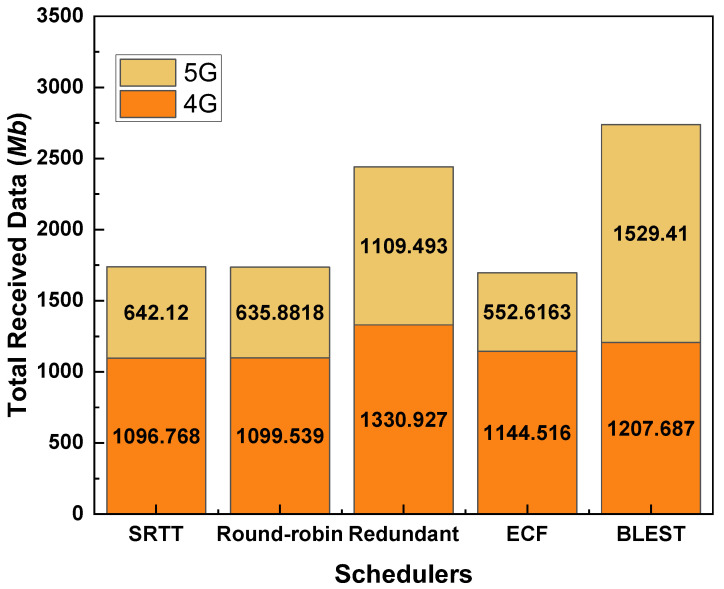
Performance of the tested MPTCP schedulers in terms of total received data for OLIA as a CCA.

The received data by the considered MPTCP schedulers while the MPTCP CCA is OLIA are shown in Figure 6. Following the results of LIA, the schedulers show a similar trend in performance. However, the total outcomes of each of them are a bit lower than the LIA case. We believe that OLIA could not adapt to the changing network scenarios like LIA. This led to slight overall performance degradation by all the schedulers.

**Figure 7:**
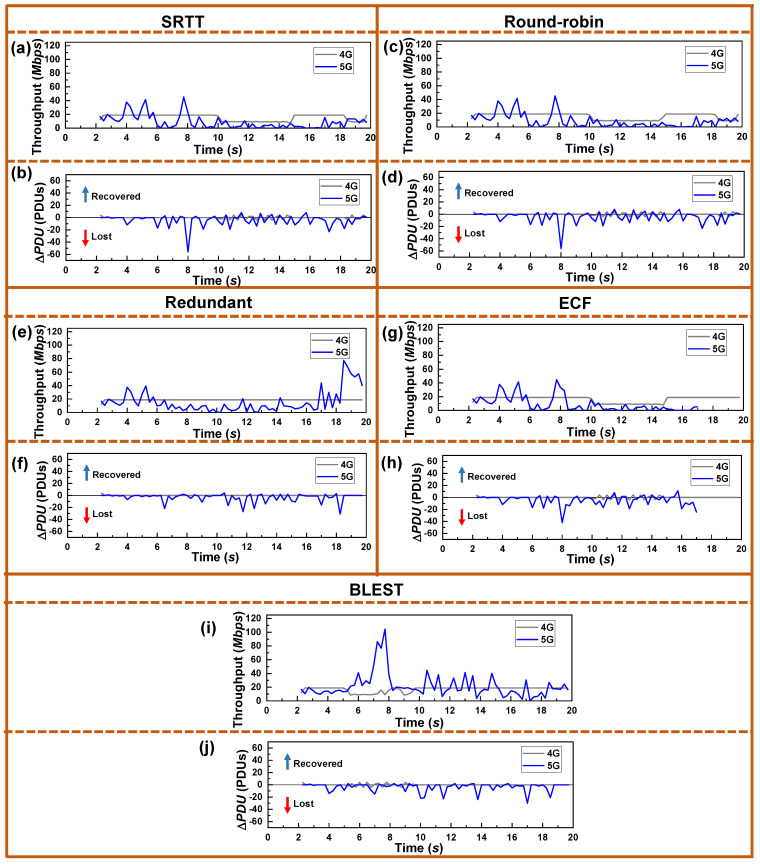
Real-time performance of the tested MPTCP schedulers for OLIA as a CCA in terms of throughput and Δ*PDU* for: (**a**,**b**) SRTT, (**c**,**d**) Round-robin, (**e**,**f**) Redundant, (**g**,**h**) ECF, and (**i**,**j**) BLEST, respectively.

Figure 7 shows the real-time throughout and Δ*PDU* obtained by the schedulers during the simulation time. The SINR was the same, as shown in Figure 3. Again, a similar trend in the result is observed as that of LIA. Like LIA, OLIA also increases the CWND of the 5G SF at 8 s. The SRTT, Round-robin, and ECF schedulers schedule the packets for the 5G SF considering the availability of CWND and lowest RTT, which are lost due to a drop in SINR and result in HoL blocking at the receiver. However, we notice a different event here, i.e., ECF stops sending through the 5G SF after 17 s. We believe that as the SINR remains low for almost 8 s by that 17-second time, ECF does not receive enough improvements in the CWND by OLIA. It leads ECF to believe that the 5G SF is no longer providing any benefit for data transmission through it.

Table 3 summarizes the FCT for this experiment. Again, it produces a similar trend with a slight increase in overall FCT, which can be mainly attributed to OLIA’s excessive fairer behavior towards SPTCP flows.

### 4.4. Performance Evaluations of MPTCP Schedulers When the CCA Is BALIA

**Figure 8:**
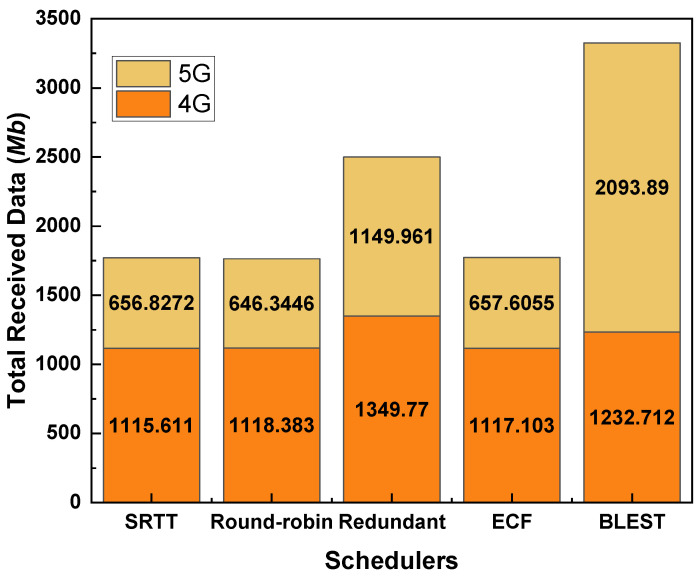
Performance of the tested MPTCP schedulers in terms of total received data for BALIA as a CCA.

The performance of the considered MPTCP schedulers in terms of total received data for BALIA as a CCA can be observed in Figure 8. As we can follow, although the other schedulers present a similar trend in performances like LIA and OLIA, they could receive the highest amount of data for BALIA. Moreover, BLEST obtains the highest received data of 2093.89 Mb in 5G. In addition to the 4G-received data of 1232.712 Mb, BLEST receives a total of 3326.602 Mb data, which is the highest among all the schedulers. Thus, BLEST in combination with BALIA, provides the best performance in the considered scenario. It can be noted that the significantly higher amount of total received data is received just in those times when the UE has enough signal strength, thus indicating the superiority of the 5G network over 4G. Moreover, this gives a glimpse of the improved performance that can be obtained by implementing a more intelligent scheduler for the combined use of 4G and 5G networks.

**Figure 9:**
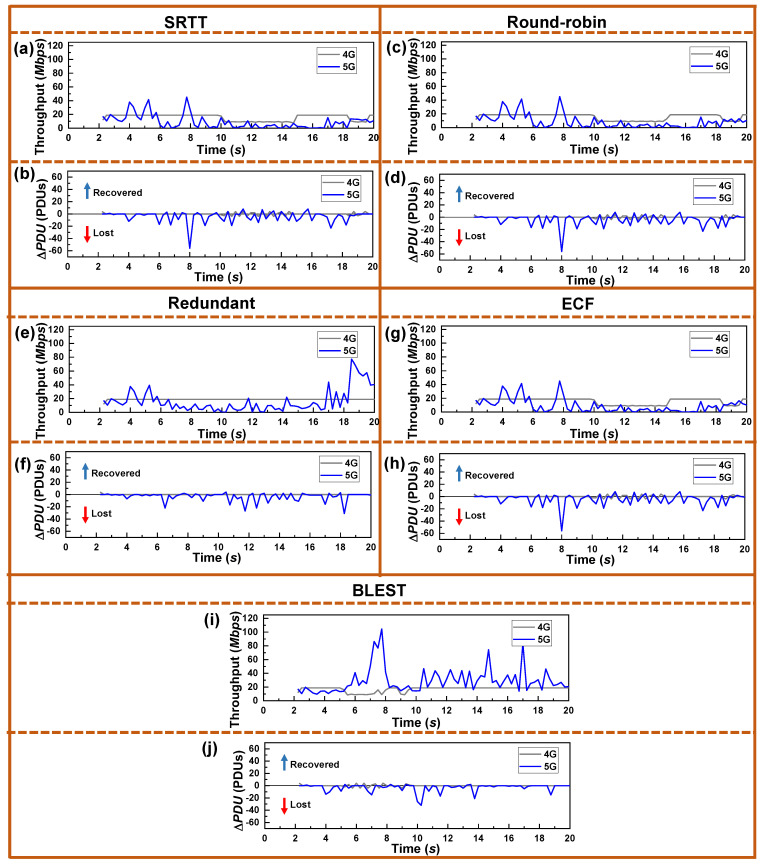
Real-time performance of the tested MPTCP schedulers for BALIA as a CCA in terms of throughput and Δ*PDU* for: (**a**,**b**) SRTT, (**c**,**d**) Round-robin, (**e**,**f**) Redundant, (**g**,**h**) ECF, and (**i**,**j**) BLEST, respectively.

The throughput and Δ*PDU* measured in real-time during the simulation experiment are given in Figure 9. Again, the SINR was the same as Figure 3. We can observe the same trend in the results as we observed for the tests in LIA and OLIA as a CCA. However, it can be noted that BLEST better utilizes the 5G SF in combination with BALIA as a CCA in this test compared to the other schedulers and CCAs. This highly contributed to BLEST obtaining the highest throughput. We believe that BALIA provides a comparatively better response to network changes than LIA and OLIA. BLEST’s intelligent scheduling algorithm takes better advantage of it and yields the highest throughput.

The FCT observed while the CCA is BALIA for the tested schedulers is listed in Table 4. Similar to the throughput result, BALIA could complete the flows at the lowest time of 6.012 ms on average. The other schedulers perform in a similar fashion, as observed previously in LIA and OLIA, but with a slightly lower FCT.

### 4.5. Performance Evaluations of MPTCP Schedulers When the CCA Is wVegas

**Figure 10:**
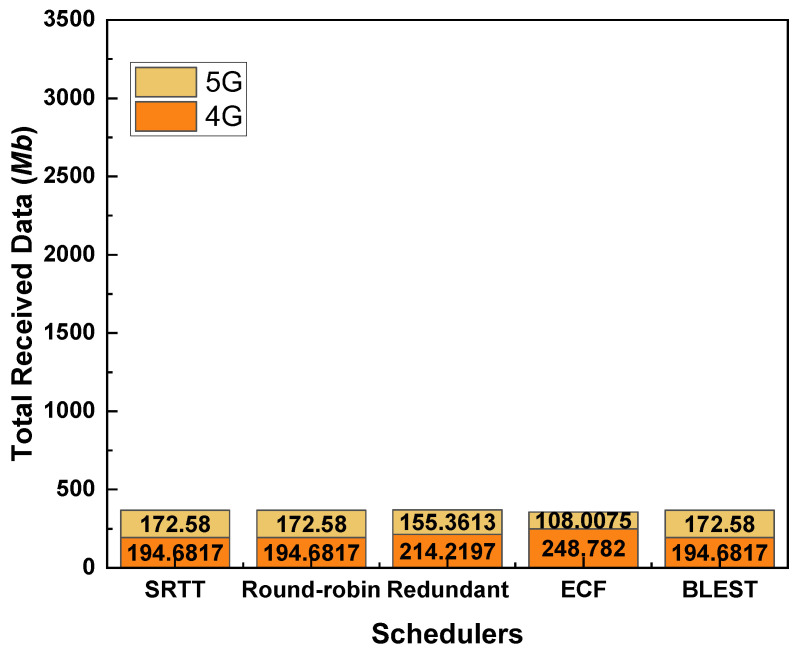
Performance of the tested MPTCP schedulers in terms of total received data for wVegas as a CCA.

Figure 10 shows the total data received by the MPTCP schedulers for wVegas as the CCA. Interestingly, significantly different from previous experiments, all the schedulers received almost the same amount of total received data in this case. As we explained previously, rather than focusing on the design goals of MPTCP, wVegas focuses on their proposed “congestion equality principle”. This limits its functionality and unnecessarily restrains its CWND from growing at times, which is the key reason that it could not utilize the available network resources properly.

**Figure 11:**
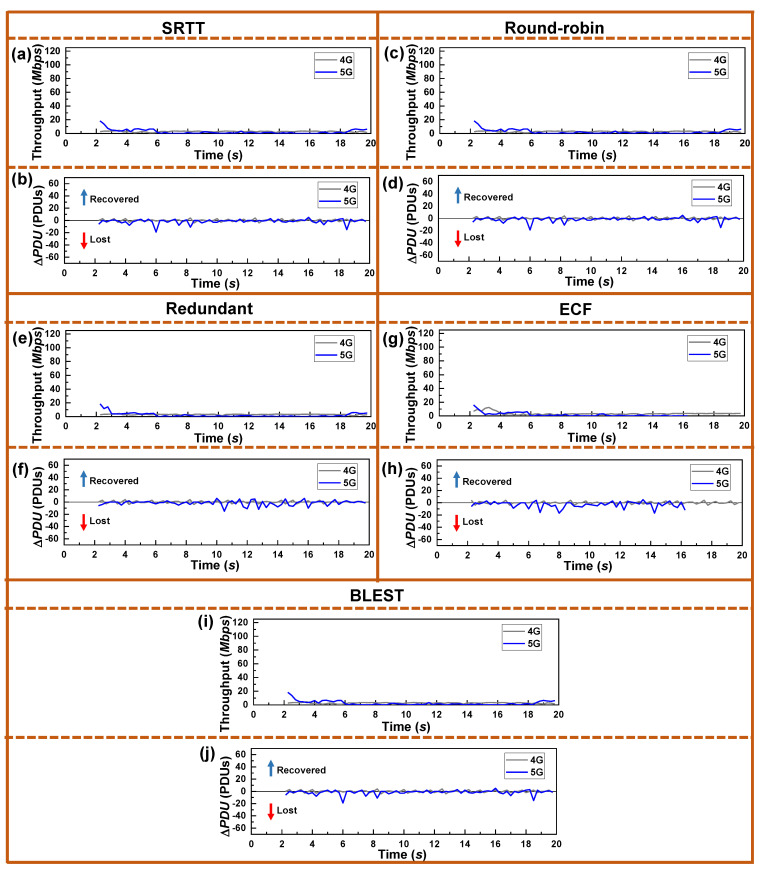
Real-time performance of the tested MPTCP schedulers for wVegas as a CCA in terms of throughput and Δ*PDU* for: (**a**,**b**) SRTT, (**c**,**d**) Round-robin, (**e**,**f**) Redundant, (**g**,**h**) ECF, and (**i**,**j**) BLEST, respectively.

Figure 11 shows recorded throughput and Δ*PDU* received by the UE in real-time during the simulation. The observed SINR was the same as shown in Figure 3. Contrary to the previous experiments, schedulers using wVegas show a greatly reduced number of packet losses. The throughput is substantially low for both the SFs in all the schedulers. This low throughput is because of wVegas’s dependency on the “congestion equality principle.” As it sends significantly less data, the network remains free. As a result, a small number of packet losses are observed.

Finally, Table 5 summarizes the FCT obtained by considered schedulers for wVegas. Similar to the low throughput results, measured FCTs are also very high for wVegas. The FCTs of all the schedulers are almost the same as each other. This indicates that the CCA, which is wVegas, dominates in this experiment. The results are due to wVegas’s dependency on the “congestion equality principle,” which restrains it from utilizing the available bandwidth.

## 5. Conclusions

In this work, aiming to improve the throughput of the 5G network deployed in an NSA setup, we focus on observing the performance and finding a better combination of MPTCP scheduler and CCA. We implemented a testbed based on NS3-DCE that enables simulating MPTCP’s performance in both 3GPP and non-3GPP networks. We also designed a scenario where a UE can transfer data through both 4G and 5G network interfaces simultaneously, and the network condition changes dynamically. We implemented Redundant, ECF, and BLEST schedulers in the testbed along with the previously available SRTT and Round-robin schedulers.

Through extensive simulation experiments, we confirm that BLEST scheduler, in combination with BALIA CCA, provides the highest throughput due to their proper adaptation to network changes. Moreover, we found a direct relation between SINR, throughput, and packet losses measured at the UE and briefly explained their relation.

In future work, we plan to enable multipath quick user datagram protocol internet connection (MPQUIC) in the NS3-DCE testbed to enable comparison of MPTCP with MPQUIC considering 5G and 4G scenarios. We further plan to design a scheduler that considers SINR, CWND, throughput, and losses to make the proper scheduling decision to ensure high throughput by reducing HoL blocking at the receiver.

## Figures and Tables

**Table 1:** A brief overview of the existing research works on the performance analysis of the MPTCP schedulers.

Research Work	Test Tool	Considered Scenario
Paasch et al. [40]	MiniNet [39]	Simple, connecting client and server via two paths
Thakur et al. [41]	Physical Testbed	Simple, connecting client and server via two paths
Sathyanarayana et al. [42]	Physical Testbed	Client and server connects via two cellular network operators
Kumar et al. [43]	Physical Testbed	Simple 5G/B5G hybrid network

**Table 2:** Performance of the tested MPTCP schedulers in terms of FCT for LIA as a CCA.

Scheduler	Flow Completion Time (FCT)
	ms
Default (SRTT)	11.476
Round-robin	11.331
Redundant	7.999
ECF	11.655
BLEST	7.219

**Table 3:** Performance of the tested MPTCP schedulers in terms of FCT for OLIA as a CCA.

Scheduler	Flow Completion Time (FCT)
	ms
Default (SRTT)	11.502
Round-robin	11.525
Redundant	8.195
ECF	11.785
BLEST	7.31

**Table 4:** Performance of the tested MPTCP schedulers in terms of FCT for BALIA as a CCA.

Scheduler	Flow Completion Time (FCT)
	ms
Default (SRTT)	11.284
Round-robin	11.333
Redundant	8.001
ECF	11.269
BLEST	6.012

**Table 5:** Performance of the tested MPTCP schedulers in terms of FCT for wVegas as a CCA.

Scheduler	Flow Completion Time (FCT)
	ms
Default (SRTT)	54.457
Round-robin	54.457
Redundant	54.115
ECF	56.055
BLEST	54.457
